# Social genetic and social environment effects on parental and helper care in a cooperatively breeding bird

**DOI:** 10.1098/rspb.2015.0689

**Published:** 2015-07-07

**Authors:** Mark James Adams, Matthew R. Robinson, Maria-Elena Mannarelli, Ben J. Hatchwell

**Affiliations:** 1 Department of Animal and Plant Sciences, University of Sheffield, Sheffield, UK; 2 Queensland Brain Institute, University of Queensland, St Lucia, Queensland, Australia

**Keywords:** indirect genetic effects, associative effects, cooperative breeding, kin selection, long-tailed tits, *Aegithalos caudatus*

## Abstract

Phenotypes expressed in a social context are not only a function of the individual, but can also be shaped by the phenotypes of social partners. These social effects may play a major role in the evolution of cooperative breeding if social partners differ in the quality of care they provide and if individual carers adjust their effort in relation to that of other carers. When applying social effects models to wild study systems, it is also important to explore sources of individual plasticity that could masquerade as social effects. We studied offspring provisioning rates of parents and helpers in a wild population of long-tailed tits *Aegithalos caudatus* using a quantitative genetic framework to identify these social effects and partition them into genetic, permanent environment and current environment components. Controlling for other effects, individuals were consistent in their provisioning effort at a given nest, but adjusted their effort based on who was in their social group, indicating the presence of social effects. However, these social effects differed between years and social contexts, indicating a current environment effect, rather than indicating a genetic or permanent environment effect. While this study reveals the importance of examining environmental and genetic sources of social effects, the framework we present is entirely general, enabling a greater understanding of potentially important social effects within any ecological population.

## 1. Introduction

Social interactions, such as competition and cooperation, are key factors in evolution by natural selection as they generate fitness differences among individuals [1–3]. However, when individuals interact, they can influence each other's phenotypes, thereby shaping the traits upon which selection acts [4,5]. The social effect of one individual on another's phenotype, also called associative or indirect effects, occurs in situations such as contest competition [6] and the coordination of parental effort [7]. Social effects may thus play a major role in the evolution of social systems [4,8–10], and therefore are important to estimate for social traits in wild populations.

One such social system, cooperative breeding, is broadly defined by more than two individuals providing care for offspring, and has evolved in a wide range of taxa [11]. Kin-selection models [1] have been used to understand the evolution of cooperation, with many studies demonstrating that indirect fitness benefits can be gained through helping relatives to reproduce [12]. However, such studies ignore the social effects [8,13,14] that could be generated by interactions within cooperative breeding groups. Cooperating individuals usually differ from each other in effort, both in the amount of parental care provided by breeding parents and in the amount of help by other members of cooperative groups. Underlying differences in effort among carers may result from various factors, including heritable variation in investment [15] and condition dependence [16]. The social effect of one individual on another could arise when parents negotiate effort with partners [17] and when they reduce their effort in the presence of helpers [18]. Such social effects are a property of an individual and estimate that individual's influence on other carers. These effects are considered to be indirect (A's effects on B's phenotype) in contrast to the direct effects (A's effects on its own phenotype).

For cooperative breeders’ provisioning behaviour, the presence and magnitude of these social effects measure the responsiveness to partners’ and helpers’ effort. If individuals maintain the same effort, regardless of the presence of other carers, investment is defined as additive among carers and there will be no social effects. If, on the other hand, investment is compensatory, with individuals adjusting their effort to maintain the same level of total care in the presence of helpers who vary in effort, social effects will exist between members of a breeding group. If the adjustment is proportional to the relative ability of a particular carer, the direct and social effects will be negatively correlated. In this case, a parent would decrease their effort less in the presence of a poor helper compared with a good helper. In contrast, matching of provisioning effort [7,19] would result in a positive correlation. The presence of a correlation between the direct and social effects also depends on whether an individual adjusts its behaviour in response to the same phenotype of its group members. For example, a parent might adjust its provisioning rate in response to the total quantity of food brought in by a helper, but not to the helper's own provisioning rate. One helper might bring back larger food items a few times per hour, whereas another helper brings smaller items many times per hour. If the two types of helper bring in the same total amount, then under this scenario, the parent would lower their effort by the same amount, but on average, the parent's response would be uncorrelated with helpers’ rates. This parental response would show up as a social effect on provisioning rates without being correlated with an individual's direct effect on their own provisioning rate.

Here, we examine both direct and social effects on parental effort in a wild, cooperatively breeding species. Using a long-term study of long-tailed tits *Aegithalos caudatus*, we use an approach of modelling social interactions derived from applied quantitative genetics [20]. The key advances from these statistical models lie in their ability to estimate variation as a sum of direct genetic and environmental effects, and the social effects of individuals with whom they interact. These social effects [4] can likewise be partitioned into genetic and environmental components [21], also called indirect genetic effects (IGEs) and indirect environment effects (IEEs). IGEs have implications for trait evolution [13], because genetic variance underlying social effects also contributes to the total heritable variance available for selection [22]. The existence of IGEs means that the genotypes of helpers influence the phenotype of the parents. Therefore, understanding the diversity of cooperative breeding in natural systems and the variation in breeder and helper investment strategies requires a complete understanding of relatedness and heritable variation linked to direct and social effects [13].

**Figure 1.**
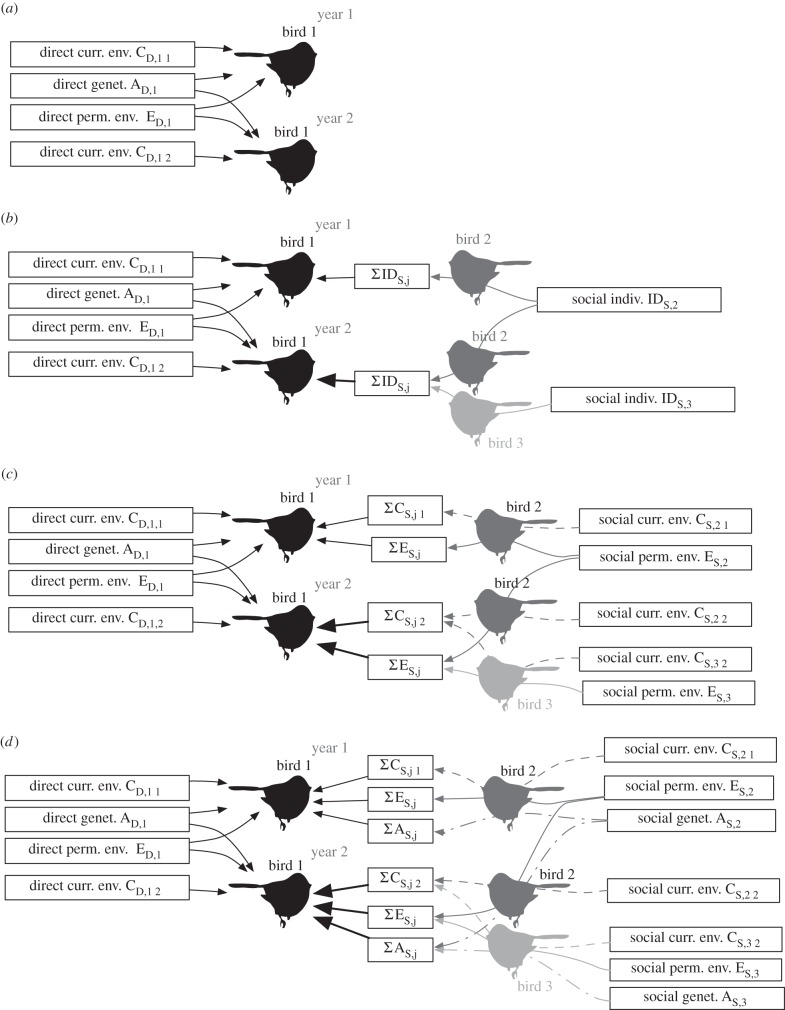
Diagram of social effects models, showing how behaviour of a focal individual (bird 1) over years 1 and 2 is modelled with direct and social effects. For clarity, fixed effects and nest effects are not visualized. (*a*) Baseline models (1A and 2A) of direct effects from focal bird 1 on its own behaviour. (*b*) Social identify effect models (1B and 2B) of the effect of bird 1's social partners (birds 2 and 3) on its behaviour. (*c*) Social environment models (1C and 2C) split social effects into permanent environment effects (consistent across years) and current environment effects (consistent within years). (*d*) Social genetic models (1D and 2D) partition permanent effects into a permanent environment component and a permanent genetic component. Curr., current; perm., permanent; genet., genetic; env., environment.

In long-tailed tits, all adults attempt to breed every year, often with different partners over the course of their lives owing to mortality and divorce [23]. Nests often fail because of high nest predation [24], and some failed breeders become helpers at the nest of another pair [25] who are usually, but not always, relatives [26,27]. The presence of helpers leads to an increase in total provisioning rate and nestling mass [28], as well as a decrease in the provisioning rate by individual parents [29,30]. From this modulation of parental effort by the presence of helpers, we hypothesized that there are social effects between parents and helpers, and that these effects are neither completely additive nor completely compensatory. To investigate social effects on caring behaviour in this species, we extended the indirect effects modelling framework [31] in two ways. First, because we had observed individuals multiple times within and across years, we were able to partition individual variance into genetic effects and two environment effects: permanent environment effects that persist over an individual's lifetime and current environment effects that differ between years (figure 1). We were thus able to estimate how much social effects varied between years as a test of whether social effects could be condition-dependent. Second, in their social groups, birds take on one of two social roles: that of parent or helper. We were able, therefore, to further partition the social environment effects into those from parents and those from helpers. Finally, a social effect is defined by behavioural plasticity, because the effect captures the responsiveness of a focal individual to the presence or behaviour of a particular social partner. However, a focal individual may also respond to other factors that change over time, such as changes in group size and brood demand. Therefore, we also investigated the relative magnitude of within-individual variation attributable to social effects and other factors.

## 2. Methods

### (a) Study system

A population of approximately 25–72 breeding pairs of long-tailed tits in the Rivelin Valley, Sheffield, UK (53°23′ N, 1°34′ W) was studied intensively from 1994 to 2011. The study site covers about 3 km^2^, and includes woodland, scrub and farmland. Birds were ringed with unique combinations of colour rings either as nestlings or as adults, after capture in mist nets (under BTO licence). In each breeding season, at least 95% of adults were colour-ringed, and the breeding attempts of all pairs in the study site were closely monitored. A small proportion of nesting attempts (probably < 5%) were not found, but the great majority of these were short-lived attempts that quickly failed [32]. Nests were checked every 2–3 days, and in the event of nest failure, we searched for re-nesting attempts. We recorded the day on which the first egg of a clutch was laid, and clutch size was determined for accessible nests once incubation started (usually on the day of clutch completion). Hatching in long-tailed tits is synchronous, and hatch date (day 0) was determined from daily nest checks from day 13 of the incubation period onwards. Following hatching, most nests were observed for recording of provisioning rates at 2-day intervals from day 2 until fledging (typically day 16 or 17) or nest failure. Nestlings were ringed and brood size recorded on day 11 of the nestling period. Blood samples (approx. 10 µl) were taken by brachial venipuncture (under UK Home Office licence) from nestlings and adults at the time of first capture. For further details of relevant field methods, see MacColl & Hatchwell [29] and Meade *et al.* [30].

We used provisioning rate (typically during a 1 h observation period) as a proxy measure of parental effort, a measure that provides a robust measure of investment [28,29]. The sample of provisioning data analysed here consisted of 344 individuals provisioning at 195 nests. There were 55 birds that were observed as both parent and helper, 206 that were observed only as parents and 83 that were observed only as helpers. In total, there were 2800 measures of visits per hour. On average, each bird was measured 8.1 times (range 1–36) across 1–6 years (mean = 1.4), and 27% of birds were sampled in more than 1 year. The mean number of birds provisioning each nest was 2.8 (range 2–7), and the median number of days each nest was observed was 7 (range 1–14).

### (b) Genotyping and pedigree construction

We extracted genomic DNA from blood samples as previously described [33], and all sampled individuals were genotyped at 19 autosomal microsatellite loci, arranged in three multiplexes that also included two sex-typing markers (electronic supplementary material, table S1). No locus deviated from Hardy–Weinberg equilibrium nor displayed linkage disequilibrium after a correction for multiple tests (electronic supplementary material, table S1). Individuals were sex-typed using the *P2D-P8* and *Z-002A* markers [34,35]. We used the microsatellite markers to assign parents to offspring and identify full-siblings in the pedigree (electronic supplementary material).

### (c) Quantitative genetic analysis

We estimated genetic and environmental effects on feeding rates (square-root transformed) using a mixed-effects animal model [21,36] implemented in ASReml [37]. We first built a baseline model (model 1A) of the direct permanent environment, direct current environment, direct genetic and nest effects on feeding rates of parents (figure 1 and table 1; electronic supplementary material). This model and subsequent models included fixed effects to capture known sources of variability: sex, age of the focal bird (years), whether helpers were related to the breeder, brood size, number of helpers, hour of day observed, age of the brood (days) and interactions of sex with brood age and number of helpers. We then tested for social effects from helpers (model 1B), tested whether social effects of helpers were consistent within years or differed between years (model 1C), and tested whether there was a genetic basis to helper social effects (model 1D; table 1). We repeated the model-building procedure using feeding rates of both parents and helpers (models 2A–D) to estimate social effects from all members of a breed group (table 1). Using models 2B–D, we also estimate correlations between direct and social effects. We also tested for dilution of the social effects where social effects attenuate in larger groups [38], because the number of individuals provisioning differed between nests.

**Table 1.** Direct and social effects and model variance components.

category	source individual	source component	relevant timespan	levels^a^	model term^b^	variance contribution^c^	interpretation
direct	carer	permanent environment	lifetime	bird ID	*E*_*D*,*i*_	*V*_PE_	non-genetic effects of a focal individual on its own phenotype that persist across all observations
	carer	current environment	year	bird-year	*C*_*D*,*i*,*y*_	*V*_CE_	effects of a focal on its own phenotype that are consistent within a breeding season but differ between years
	carer	genetic	lifetime	pedigree	*A*_*D*,*i*_	*V*_A_	focal individuals genes’ effect on its own phenotype
	carer	residual	day		*E_i_*_*yk*_	*V*_R_	day-to-day variability in a focal individual's effort after accounting for all other effects
social	helper	identity	lifetime	bird ID	ID_*S,h*_	*H*^1−2*d*^ *V*_ID(H)_	average social effect of a helper on all the breeding pairs it helps; term includes contribution from genetic and individual environment factors; total contribution to the variance in the targets’ (recipients’) phenotypes is a function of the average number of helpers *H*, which may be diluted (*d*) as the number of helpers increases; if there is no total helper social effect variance, *V*_ID(H)_ = 0, it means that parents respond generically rather than to specific qualities of their helpers
	helper	permanent environment	lifetime	bird ID	*E*_*S,h*_	*H*^1−2*d*^ *V*_PE(H)_	average non-genetic effect a helper has on all breeding pairs it interacts with
	helper	current environment	year	bird-year	*C*_*S,h,y*_	*H*^1−2*d*^ *V*_CE(H)_	social effects from helpers that vary between years
	helper	genetic	lifetime	pedigree	*A*_*S,h*_	*H*^1−2*d*^ *V*_A(H)_	influence of a helper's genes on the provisioning rate of the parents
social	parent or helper	identity	lifetime	bird ID	ID_*S*,*j*_	*J*^1−2*d*^ V_ID(S)_	a bird's average influence on members of every breed group that it is part of; total variance is a function of the average number of social partners (mate + helpers) in every breed group, *J,* which may become diluted (*d*) in larger groups
	parent or helper	permanent environment	lifetime	bird ID	*E*_*S*,*j*_	*J*^1−2*d*^ *V*_PE(S)_	a bird's average non-genetic influence on all its social partners
	parent or helper	current environment	year	bird-year	*C*_*S*,*j*y_	*J*^1−2*d*^ *V*_CE(S)_	a bird's average influence on its social partners within a given year
	parent or helper	genetic	lifetime	pedigree	*A*_*S*,*j*_	*J*^1−2*d*^ *V*_A(S)_	average genetic effects of a bird on all of its social partners
shared		nest	year	nest ID	*N*_*m,y*_	*V*_N_	similarity in provisioning rate of all members of a breed group
composite	carer	consistency	year	bird-year		*V*_CST_	sum of all variance components except residual variance; expresses the consistency (intraclass correlation) of a bird's feeding rate within a given year after accounting for fixed effects
	carer	adjusted phenotypic	day			*V*_CST_ + *V*_R_	total phenotypic variance after accounting for fixed effects

^a^Levels used to specify random effect in model.

^b^Term subscripts: *D,* direct effect; *S,* social effect. Index subscripts: *i,* focal bird (source and target of direct effects, target of social effects); *h,* helper (source of helper social effects); *j,* parent or helper (source of social effects); *m,* nest; *y,* year; *k,* day.

^c^*H,* average number of helpers; *J,* average number of social partners; *d,* dilution parameter.

We estimated how consistent birds were in their provisioning effort after accounting for known environmental factors prompting behavioural plasticity, such as brood age. The ratio between the consistency variance (*V*_CST_) and the adjusted phenotypic variance (*V*_P_) is an intraclass correlation coefficient (ICC) and equals the expected correlation between a bird's feeding rate on separate days in the same year after accounting for fixed effects, equivalent to an ICC(3,1) [39]. This is the appropriate scale on which to compare the magnitude of variance from social effects because it removes variance from other factors that would make a parent's feeding rate differ before and after a helper joins the nest. We also estimated adjusted heritability [40] on this scale as it renders an estimate that is comparable with previous studies that used average feeding rates [15].

We used the likelihood ratio (LR) test to assess the statistical significance of adding social variance components and weighted AIC to assess relative fit across models. The total contributions of social effect variance were adjusted by average group size and dilution effects [31,38]. Because the parameters of interest were functions of multiple variance components, we generated confidence intervals for model parameters by bootstrapping residuals (electronic supplementary material). Finally, we conducted a sensitivity analysis to check our ability to estimate social effects from our data; tested whether direct effect variance differed by sex, breed role or group size; tested whether social effects differed by target or partner sex; and examined phenotypic plasticity to time-varying factors (brood age and number of helpers) as a possible confound (electronic supplementary material).

## 3. Results

**Figure 2.**
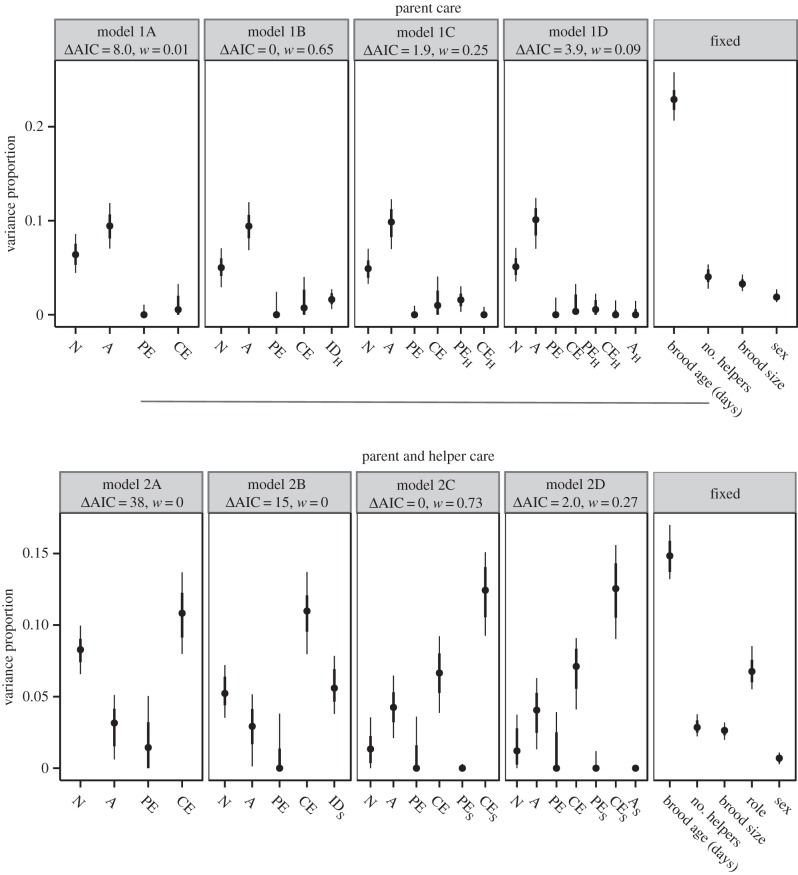
Effect sizes. Variance proportions for fixed and random effects predictors relative to observed phenotypic variance *V*_P_. Point estimates are surrounded by 50% (heavy lines) and 80% (thin lines) confidence intervals calculated from parametric bootstrapping. Model 1A fit to parent phenotype; model 2A fit to parent and helper phenotype. Models 1A and 2A include only direct effects; models 1B and 2B add social effect of partner identity; models 1C and 2C split social effect into permanent environment and current environment; models 1D and 2D fit social genetic effects. Variance components: *A,* additive genetic; CE, current environment; PE, permanent environment; *N,* shared nest environment; ID, social partner identity. H and S subscripts denote social effects from helpers or all breed group members, respectively. Variance attributable to social effects were obtained by multiplying fitted variances by average number of social partners. ΔAIC gives difference between model fit and that of the best model and *w* gives the AIC weight.

We first estimated the direct environmental and genetic effects on provisioning rate of parents and helpers (table 1). Given that provisioning rate needs to be an extremely plastic trait to respond to changes in brood demand and load sharing with other carers, individuals were moderately consistent in their feeding rate across days at a nest within a particular year (*V*_CST_/*V*_P_ = 0.24, CI = 0.21, 0.27). Most of the consistency in provisioning rates was from differences between nests (*V*_N_/*V*_CST_ = 0.34, CI = 0.25, 0.43), and each bird's current environment effect (*V*_CE_/*V*_CST_ = 0.44, CI = 0.27, 0.61). Permanent environment (*V*_PE_/*V*_CST_ = 0.08, CI = 0.00, 0.29) and additive genetic effects (*V*_A_/*V*_CST_ = 0.12, CI = 0.00, 0.29) together made up less than one-third of the within-year variance ((*V*_A_ + *V*_PE_)/*V*_CST_ = 0.20, CI = 0.07, 0.37). Variance components as a proportion of the observed phenotypic variance are plotted under model 2A in figure 2. Heritability, as a proportion of a bird's mean feeding rate each year, was higher when considering only the effort of birds when they were parents (*V*_A_/*V*_CST_ = 0.55, CI = 0.40, 0.73; model 1A; figure 2). We did not find any evidence for a sex difference in direct effects (electronic supplementary material).

We then estimated social effects on provisioning rate while accounting for the direct effects described above. Because the composition of breeding groups in our study population was fluid between and within years, we were able to estimate two types of social environment effects in addition to social genetic effects (figure 1 and table 1). The first was a social permanent environment effect that captures the average deviation in feeding rate of all the birds that provision the same nests that a focal bird does across its lifetime. The second was a social current environment effect. This effect is temporary and restricted to a given year, and captures the average deviation in feeding rates of individuals who are provisioning a particular nest at the same time as a focal carer.

On average, across all individuals, feeding rate increased when helping a relative, and with increasing brood size and age (electronic supplementary material). Helpers had lower feeding rates than parents, and the feeding rate of both categories of carers decreased when more helpers were present (electronic supplementary material). These findings are consistent with previous studies on this system [27,29,30,41].

At the individual level, helpers differed in the social effects they had on parents within a given year (LR = 10.0, d.f. = 1, *p* < 0.001; model 1B; figure 2), demonstrating individual-level social effects; in other words, the effect of a helper on parental effort in this population is dependent upon the identity of the individual helping. Social effects from helper identity accounted for a substantial portion of the repeatable variance in parental feeding rates (*H*V_ID(S)_/*V*_CST_ = 0.20, CI = 0.09, 0.32). There was no evidence of dilution of social environment helper effects on parents as helper number increased (*d* = 0.0; electronic supplementary material), suggesting that the effect of any one individual on another does not weaken with increasing group size. We were not able to separate out the relative contributions of permanent and current environment sources of helper social effects (model 1C; figure 2*a*). Interestingly, we found no detectable social genetic effects of helpers on parents (LR = 0.7, d.f. = 1, *p* = 0.79) at a 5% significance level when compared with a model that included social environment effects, and the total contribution of helper social genetic effects on parental care was small (*HV_A(S)_*/*V*_CST_ = 0.05, CI = 0.00, 0.16).

Social environment effects were also significant when effects of parents on helpers and helpers on each other were considered (LR = 24.2, d.f. = 1, *p* < 0.001; model 2B; figure 2). When this social environment effect is split into its permanent and current environment components (model 2C; figure 2), the social permanent environment variance dropped to zero, where the social current environment effects explained around a quarter of the repeatable variance (*J*^(1 − 2*d*)^*V*_CE(S)_/*V*_CST_ = 0.21, CI = 0.11, 0.30). There was some dilution of the social current environment effects as group size increased (*d* = 0.1; electronic supplementary material). Much of the within-individual, between-year variation in provisioning rate can be assigned to effects from a bird's current social environment. While the correlation between direct and social current environment effects was not statistically significant (LR = 1.8, d.f. = 1, *p* = 0.18), the direction of the correlation was negative (*r*_CE_ = −0.25, CI = −0.94, −0.13), indicating that members of a social group are responsive to each other's presence, and suggesting that the response is compensatory. As in our previous analysis of helper social effects, there were no detectable social genetic effects (LR = 0.00, d.f. = 1, *p* = 1) of birds (either parents or helpers) on the members of the same breeding group and the estimate indicated it accounted for at most 10% of the between-individual variance (*JV*_A(S)_/*V*_CST_ = 0.02, CI = 0.00, 0.11).

Overall, this analysis of social effects of all group members on each other has revealed that helpers, as well as parents, adjust their feeding rates in response to the presence of other individuals. However, our results suggest that there is little repeatability in social effects across years, probably because of within-individual variation from changes in breeding roles and variability in condition across years. Therefore, social current environment effects (rather than permanent environment or social genetic effects) appear to be responsible for much of the variation in social breeding behaviour in this system.

We also tested for several extensions to and confounds for social environment effects. We did not find any evidence that social effects varied depending on the sex of the focal individual or of its partners, and nor did we find any evidence that social effects differed between kin and non-kin. Birds did show individual phenotypic plasticity in response to brood age, but this did not explain the significant contribution of social environment effects to feeding rates (electronic supplementary material). Thus, we can rule out at least some non-social factors, which otherwise have extremely large effects on parental care, as spuriously creating social effects.

Finally, the size of the direct and social current environment effects was large compared with most of the fixed effects (figure 2). For example, the proportion of phenotypic variance explained by social current environment effects (i.e. the total effect on an individual's behaviour from all its social partners within a given year) was almost as large as the variance in feeding rate explained by brood age. Therefore, we have been able to demonstrate that environmentally dependent indirect social effects play a substantial role in this cooperative breeding system. We also support our results through extensive simulation, finding no evidence of systematic bias creating these social environment effects (electronic supplementary material).

## 4. Discussion

The response of carers to the provisioning behaviour of others has been extensively studied theoretically [17] and empirically [18,42], but here we examined compensatory/additive effects at an individual level to test how consistent social effects were across breeding seasons and whether social effects increased the genetic variance available for selection.

We demonstrated the presence of social environment effects within this population, meaning that individual long-tailed tits vary in helping effort and that this has an influence on the care provided by parents. Furthermore, we showed that sharing of provisioning is not completely additive, because, as the social environment effects indicate, individuals adjust their effort in response to other individuals. While the correlation between direct and social effects was not significant, its negative direction indicates a compensatory response (i.e. birds decrease their own effort in response to above average care from social partners), consistent with the load-lightening effect of helpers that has been observed previously in this population [30]. Helpers may gain indirect fitness benefits through the increased survival of related breeders resulting from this compensatory reduction of effort [30,43]. Therefore, this study supports previous conclusions that indirect fitness benefits resulting from direct kin interactions provide a compelling argument for the evolution of helping behaviour in this species. In addition, we have ruled out individual differences in responsivity to kin and non-kin helpers and individual differences in plasticity to brood demands as factors that could masquerade as social effects.

Although helpers significantly influence the feeding behaviour of parents, supporting previous studies [29,44], we find no evidence for IGEs within this population, though we acknowledge that our individual and group sample sizes are underpowered to detect them [45]. However, if they are present, our data were consistent with social genetic effects explaining no more than 10% of birds’ average performance in a given year. This is not surprising as survival in this population is low [46], limiting the potential for the repeatability of indirect effects across years. Several studies have reported that parental care is repeatable across breeding attempts [47,48], but a heritable component of provisioning effort or cooperative behaviour has rarely been shown in wild populations (for exceptions, see [15,49]). Our estimate of the heritability of mean parental feeding rate (*h*^2^ = 0.55) was consistent with that of total feeding rate in a previous study of this population (*h*^2^ = 0.43 [15]).

The social environmental effect found here is likely to reflect a bird's condition in a given year and its interaction with the nest environment. Thus, for those individuals that do help, variation in condition is likely to influence the rate at which carers provision nestlings. This interpretation is consistent with the idea that the decision of whether to become a helper or not depends on condition [41,50]. Furthermore, the effect of load-lightening on male breeder survival in the presence of helpers [30] provides further evidence for a link between care and condition in this system. There is limited evidence from other cooperatively breeding species for condition-dependent helping [51–54], but it is likely that this is a more general phenomenon [50].

In conclusion, we have empirically demonstrated the importance of examining social interactions in wild populations within a social effects framework. Applying this approach to a wild cooperatively breeding population (i) provides estimates of how individual-level variation in helping behaviour shapes parental care, (ii) allows this variation to be decomposed into environmental and genetic effects, and (iii) allows the genetic and environmental covariance to be estimated between the efforts of helpers and recipients. Importantly, we demonstrate that social effects that vary between years are a substantial source of phenotypic variance in social breeding systems. The framework we use here is completely general and will provide a new avenue for investigating social interactions within wild populations.

## Supplementary Material

Supplementary Information

## Data accessibility

Data are deposited in the Dryad repository (http://dx.doi.org/10.5061/dryad.9nj6s).
