# Supplementary material for: Social genetic and social environment effects on parental and helper care in a cooperatively breeding bird
Source: Proc Biol Sci. 2015 Jul 7;282(1810):20150689. doi: 10.1098/rspb.2015.0689 (PMC4590478; doi:10.1098/rspb.2015.0689)
Supplement: Supplementary Information [file rspb20150689supp1.pdf]

SUPPLEMENTARY INFORMATION

Social genetic and social environment effects on parental and helper care in a cooperatively breeding bird

M J Adams, M R Robinson, M-E Mannarelli, B J Hatchwell

Contents

1 DIRECT AND SOCIAL EFFECT MODELS 1
2 MODEL SPECIFICATION FOR INDIRECT GENETIC EFFECTS 4
3 DILUTION OF INDIRECT GENETIC EFFECTS 4
4 EXPRESSING PARAMETER UNCERTAINTY AND EFFECT SIZES 6
5 VARIANCE COMPONENT ESTIMATES 7
6 FIXED EFFECT ESTIMATES 7
7 SENSITIVITY ANALYSIS 10
8 SEX DIFFERENCES IN REPEATABILITY 12
9 SEX DIFFERENCES IN SOCIAL EFFECTS 13
10 EFFECT OF RELATEDNESS ON SOCIAL EFFECTS 16
11 PHENOTYPIC PLASTICITY AS POSSIBLE CONFOUND 16
12 GENOTYPING 17
13 PEDIGREE CONSTRUCTION 18

1 DIRECT AND SOCIAL EFFECT MODELS

The model for individual i's feeding rate z on measurement occasion k at nest m in year y was

$$z_{iyk} = \text{fixed effects} + E_{D,i} + C_{D,iy} + A_{D,i} + N_{my} + E_{iyk} \quad \text{(Model 1A and 2A)}$$

The model included two direct environment effects. The first was an effect of bird identity ($E_{D,i}$) to estimate permanent environment variance (V_{PE}) that captures each individual's average deviation from the population mean across all observations. The second direct environment effect was bird-year ID ($C_{D,iy}$) that fits current (within-year) environment variance (V_{CE}) and that estimates the deviation of a bird's average feeding rate in a given year from its average

feeding rate across its lifetime. Direct genetic effects $A_{D,i}$were modelled as additive genetic variance (V_A) using the additive genetic relationship matrix derived from the pedigree. We fitted ID of the nest m that the bird was provisioning in year y (N_{my}) to estimate shared environment variance (V_N), which captures the similarity in feeding rates of birds provisioning the same nest. We included fixed effects to capture known sources of variability: sex, age of the focal bird (years), breeding role (coded parent = -1, helper = 1), whether helpers were related to the breeder [1] (coded as helper not related = -1, helper related = 1, parent = 0), brood size, number of helpers, hour of day observed, age of the brood (days), and interactions of sex with role, brood age, and number of helpers. The fixed effects thus contained the constant effects of having helpers and of changes in brood demand that have the same influence on all individuals.

All fixed effects were mean centred to handle missing random effects predictors from differing group sizes. The within-individual, daily fluctuations in feeding rate (E_{iyk}) not captured by other effects defined the residual variance (V_R). We calculated heritability, repeatability, consistency, and other intraclass correlations in two ways [2]. The first, repeatability, was as a proportion of the observed phenotypic variance V_P to understand the contribution of social effects relative to the phenotypic variance attributable to known fixed effects. Because our focus was on individual differences in provisioning rate, the second way, consistency, was in proportion to the within-year variance (after removing within-individual variability between observation days within a given year and variance from known fixed effects: $V_{CST} = V_P - V_R - V_{\text{fixed}}$. Within-year consistency variance can be interpreted as variance in birds' mean effort over each year.

To compare results with a previous study of heritability in a subset of data in this sample [3] we also estimated direct effects only on parental provisioning rates. We calculated the variance attributable to fixed effects by multiplying each fixed effects coefficient by the relevant columns from the data, then calculating the variance of the resulting vector [4].

We next estimated social genetic and environment effects on feeding rate [5]. We conducted two sets of analysis: (1) using feeding rates of parents only to assess the social effects of helpers on parental performance and (2) using feeding rates of parents and helpers to assess the social effects of all members of a breeding group on each other. We first expanded the models to estimate the environment effects of helpers on parents to determine whether parents adjusted their feeding rates depending on who was helping in a given year:

$$z_{iyk} = \text{fixed effects} + E_{D,i} + C_{D,iy} + A_{D,i} + \sum ID_{S,h} + N_{my} + E_{iyk} \quad \text{(Model 1B)}$$

We fitted the social effects of each helper h ($ID_{S,h}$) by specifying an overlay design matrix (i.e., the same random effect is attached to multiple columns in the data; because the maximum number of helpers was 5, the effect was associated with 5 random predictors; see Section 2) using the helpers' IDs, which estimates variance from individual social effects ($V_{ID(H)}$). Because groups differed in the number of helpers we used the 'include' option so that missing helper predictors became the reference level and we centred the fixed-effect inputs to match this parameterization. This model tests the average effect an individual has over its lifetime on the parents that it helps.

The next model split the helper social effects into effects that were consistent across all observations of a bird ($E_{S,h}$) from effects that differed across years ($C_{S,hy}$). Consistent social effects estimated the social permanent environment variance ($V_{PE(H)}$), though this parameter would include genetic effects as well, and social effects that varied between years made up the social current environment variance ($V_{CE(H)}$). This model tests whether the social effects of a helper are consistent across breeding seasons:

$$z_{iyk} = \text{fixed effects} + E_{D,i} + C_{D,iy} + A_{D,i} + \sum E_{S,h} + \sum C_{S,hy} + N_{my} + E_{iyk} \quad \text{(Model 1C)}$$

The final model estimated the social genetic variance ($V_{A(H)}$) from social genetic effects ($A_{S,h}$). This model tests whether there is a genetic basis to the social effects of helpers that would increase the total heritability of provisioning behaviour [6]:

$$z_{iyk} = \text{fixed effects} + E_{D,i} + C_{D,iy} + A_{D,i} + \sum E_{S,h} + \sum C_{S,hy} + \sum A_{S,h} + N_{my} + E_{iyk} \quad \text{(Model 1D)}$$

We then repeated the model building procedure using data on both parent and helper phenotypes to test for social permanent environment, social current (within-year) environment, and social genetic effects of parents and helpers on the other carers provisioning the same nest. This also allowed us to test for correlations between direct and social effects. The models estimated separate contributions of social effects from each parent or helper j

$$z_{iyk} = \text{fixed effects} + E_{D,i} + C_{D,iy} + A_{D,i} + \sum ID_{S,j} + N_{my} + E_{iyk} \quad \text{(Model 2B)}$$

$$z_{iyk} = \text{fixed effects} + E_{D,i} + C_{D,iy} + A_{D,i} + \sum E_{S,j} + \sum C_{S,jy} + N_{my} + E_{iyk} \quad \text{(Model 2C)}$$

$$z_{iyk} = \text{fixed effects} + E_{D,i} + C_{D,iy} + A_{D,i} + \sum E_{S,j} + \sum C_{S,jy} + \sum A_{S,j} + N_{my} + E_{iyk} \quad \text{(Model 2D)}$$

to estimate additional permanent environment ($V_{PE(S)}$), current environment ($V_{CE(S)}$), and genetic ($V_{A(S)}$) variance components for breeding group members' social effects.

2 MODEL SPECIFICATION FOR INDIRECT GENETIC EFFECTS

Indirect effect of helper within-year effects on parent feeding rates. Overlay design specified with the `and()` syntax. Differing group sizes means that some cells in the `helper_yearN` columns are blank, which required mean centering the fixed effects.

```
asreml(sqrt(visits_per_hour) ~ 1 + male.cc + age +
                    brood_size + helper_count +
                    hour + age_days +
                    age_days:male.cc +
                    helper_count:male.cc,
                   random= ~ ide(bird_id) +
                            bird_year +
                            helper_year1 + and(helper_year2) +
and(helper_year3) + and(helper_year4) + and(helper_year5) +
                            nest_id +
                            ped(bird_id),
                   data=Feed_parents,
                   na.method.X='include',
                   equate.levels=c('helper_year1', 'helper_year2',
'helper_year3', 'helper_year4', 'helper_year5'),
                   ginverse=list(bird_id=Lotti.ainv))
```

3 DILUTION OF INDIRECT GENETIC EFFECTS

To test for attenuation of social effects with group size [7], we varied a dilution parameter *d* between 0 and 1 in increments of 0.1 and chose the

parameter value that yielded the lowest AIC. The model set up is to multiply indirect effects by a dilution factor w = (mean(helper_count) / helper_count)^d.

```
asreml(sqrt(visits_per_hour) ~ 1 + male.cc + age +
                    brood_size + helper_count +
                    hour + age_days +
                    age_days:male.cc +
                    helper_count:male.cc,
                   random= ~ ide(bird_id) +
                            bird_year +
                            w:helper_year1 + and(w:helper_year2) +
and(w:helper_year3) + and(w:helper_year4) + and(w:helper_year5) +
                            breed_group +
                            ped(bird_id),
                   data=Feed_parents,
                   na.method.X='include',
                   equate.levels=c('helper_year1', 'helper_year2',
'helper_year3', 'helper_year4', 'helper_year5'),
                   ginverse=list(bird_id=Lotti.ainv))
```

Figure S2.1. Model fit for varying values of *d*. Helper current-environment effects

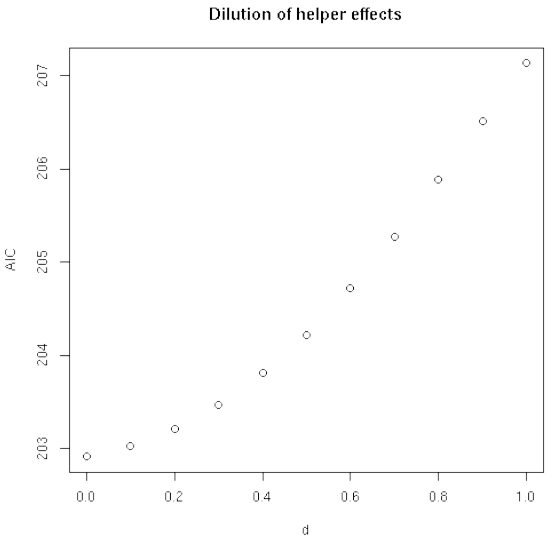

Figure S2.2. Current environment effects (breed group)

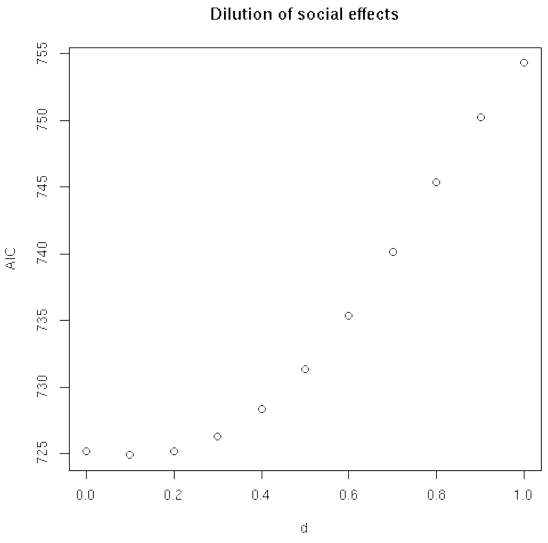

4 EXPRESSING PARAMETER UNCERTAINTY AND EFFECT SIZES

We generated confidence intervals by bootstrapping residuals. That is, a model was fitted to the data, replicate data sets (1000) were created by re-adding a residual deviance (simulated from a normal distribution parameterized with mean zero and the residual variance from the model) to the fitted value of each data point, and the model was refitted to the replicate data. We compared the effect sizes of the fixed and random effects by calculating the standard deviation (SD) of the coefficients [4]. For the fixed effects we multiplied the fitted coefficients by their associated predictor values for each row in the data, then calculated the standard deviation of the resulting columns. For the random effects we took the square roots of the variance component estimates. SDs of coefficients were transformed back to the observed scale of visits/hour as $(\theta+ \mu)^2- \mu^2$, where θ is the SD of the coefficient and μ is the model intercept. The SDs of coefficients put the size of the fixed and random effects on the same scale and can be interpreted as their relative contributions to phenotypic variance. We summarized bootstrapped parameters using means and upper and lower 95% quantiles.

5 VARIANCE COMPONENT ESTIMATES

Table S1. Variance component estimates from models. Subscripts: N = nest, A = additive genetic, PE = permanent environment, CE = current environment; ID(S) = social partner; PE(S) = social permanent environment, CE(S) = social current environment effect, A(S) = social additive genetic; R = residual, P = observed phenotypic. Social effects variances have been multiplied by average group size (see Main Text).

Model	V_N	V_A	V_{PE}	V_{CE}	$V_{ID(S)}$	$V_{PE(S)}$	$V_{CE(S)}$	$V_{A(S)}$	V_R	V_P
1A	0.048	0.070	0.000	0.004					0.347	0.745
1B	0.037	0.070	0.000	0.005	0.012				0.336	0.745
1C	0.037	0.073	0.000	0.007		0.012	0.000		0.337	0.745
1D	0.038	0.075	0.000	0.003		0.004	0.000	0.000	0.337	0.745
2A	0.067	0.026	0.012	0.088					0.359	0.809
2B	0.042	0.024	0.000	0.089	0.045				0.348	0.809
2C	0.011	0.034	0.000	0.054		0.000	0.101		0.345	0.809
2D	0.010	0.033	0.000	0.058		0.000	0.102	0.000	0.344	0.809

6 FIXED EFFECT ESTIMATES

Figure S5.1 Fixed effects coefficients from a model including random effects for nest; direct genetic, permanent environment, and current environment; and social current environment using data on both parents and helpers. Confidence intervals calculated from model-based bootstrapping (see main text). Dark lines = 50% confidence intervals, light lines = 95% confidence intervals from bootstrapping residuals.

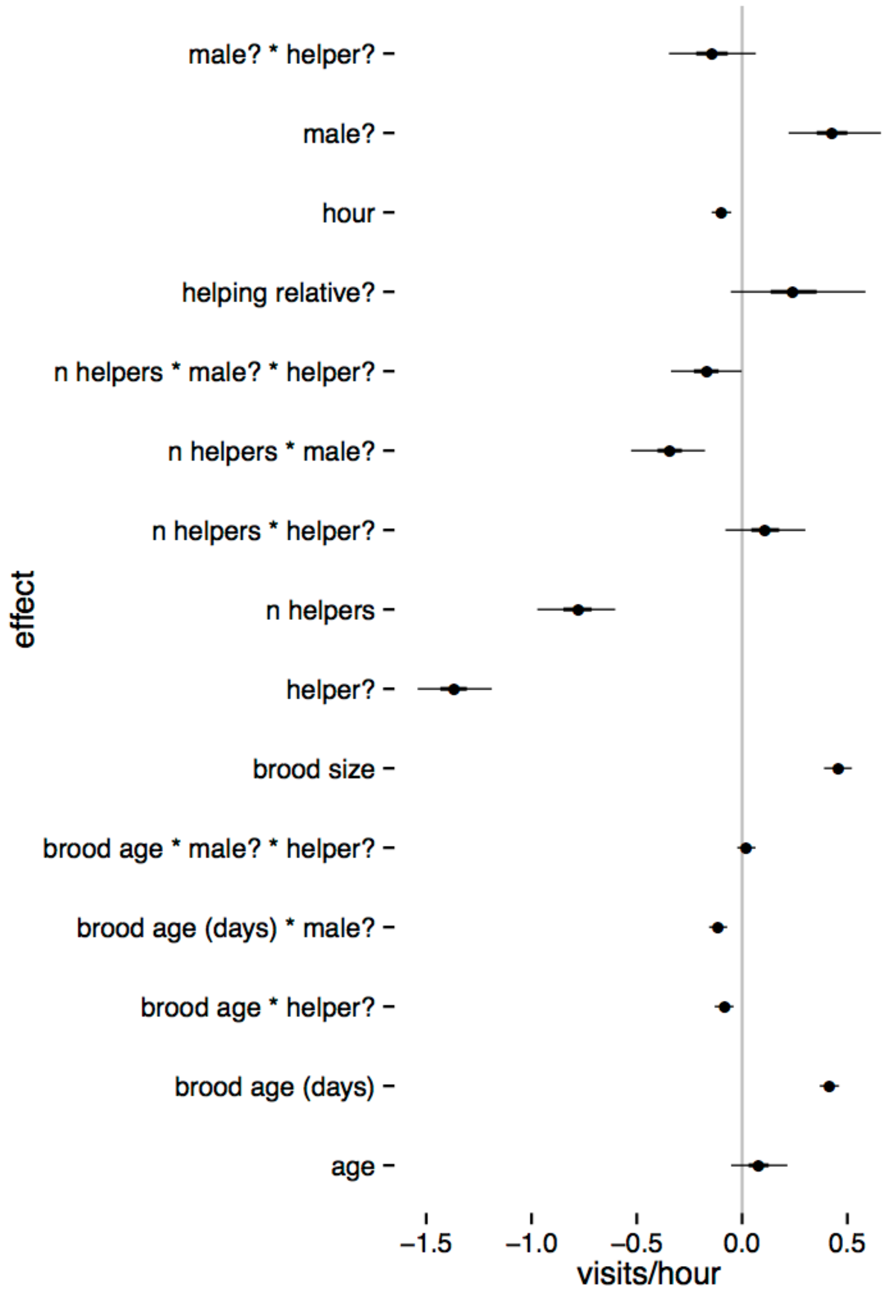

male? * helper?
male?
hour
helping relative?
n helpers * male? * helper?
n helpers * male?
n helpers * helper?
n helpers
helper?
brood size
brood age * male? * helper?
brood age (days) * male?
brood age * helper?
brood age (days)
age
effect
−1.5
−1.0
−0.5
0.0
0.5
visits/hour

Figure S5.2 Relationships between number of helpers (parents = dashed line, helpers = solid line), genetic relatedness between a helper and the parent, and brood age in days, and, size of the brood and number of provisioning visits/hour.

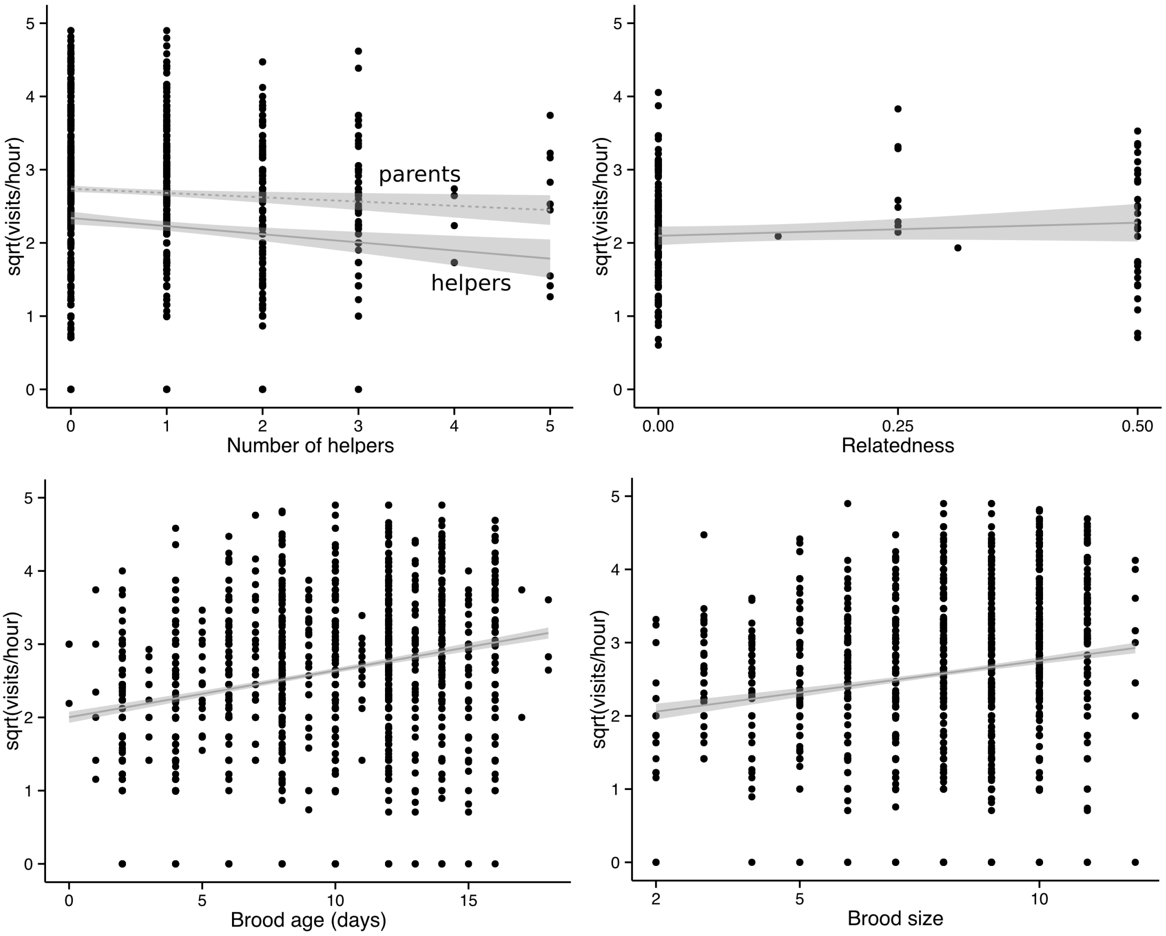

7 SENSITIVITY ANALYSIS

We assessed whether indirect effects could appear as an artefact of the data even if they were not really present. For example, individuals who fail to breed may be of lower quality and put less effort into foraging. When they join a new nest as a helper, their low foraging rate could look like an indirect effect. This could also artificially create correlations between direct and indirect effects. Likewise, direct and indirect within-year environment effects will be confounded if there are an insufficient number of observations before or after a particular individual joins a nest. To assess these possibilities, we first fitted a model that contained only direct effects to the real data. We then created simulated data sets using parametric bootstrapping. We kept the inputs for the fixed effects constant across replicates. For the random effects, we simulated a draw for each level of the effect from a normal distribution parameterized with mean zero and variance from the fitted variance component estimate. For genetic effects we simulated breeding values down the pedigree [8]. Next we fitted a model with indirect effects to the replicated data. We used the likelihood ratio test to assess the probability of mistakenly accepting the alternative (indirect effects) model (Type I error). We also compared the fitted variances and covariances for the indirect effects fitted to the observed data to the null distributions created by resampling residuals procedure.

We examined the distribution of indirect within-year environment effects in data that was simulated without indirect effects, using both parents and helpers. The indirect effects model was only accepted as better (at $\alpha = .05$) than the direct effects model 2% of the time. This was because the difference in log likelihoods between the models was sometimes negative, so the p-values were skewed towards 1. Thus if there were no indirect effects, we would be very unlikely to mistakenly accept a model that included them. The estimate for the indirect bird-year environment variance fell outside of the null distribution from simulations. There was thus no evidence of systematic bias creating these indirect environment effects.

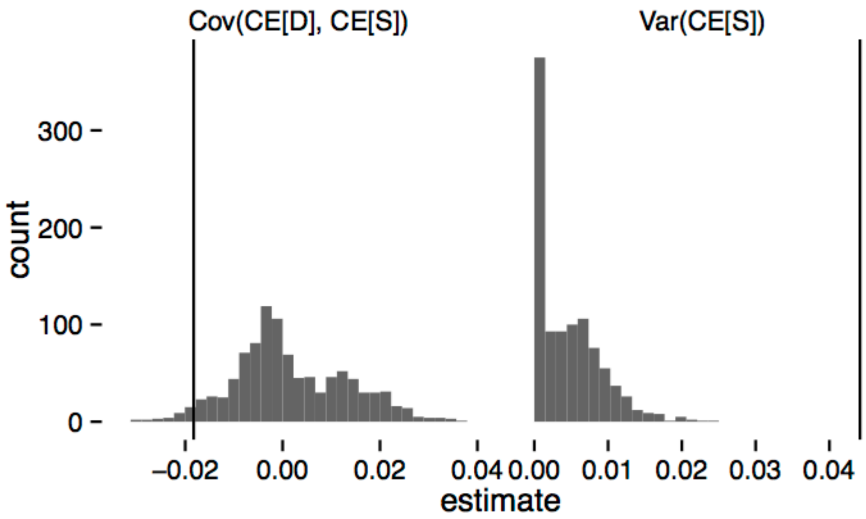

Figure S6 Sensitivity analysis for indirect bird-year environment variance, Var(CE[S]) and direct-indirect covariance, Cov(CE[D], CE[S]). Histograms are null distributions of an indirect effects model fit to data replicated by resampling from a direct-effects-only model. The point estimates from the indirect effects fit to the actual data are plotted as vertical lines.

Next we considered whether social effects could be explained by plasticity to group size or breeding role. For example, birds may differ in how much they reduce their effort in the presence of more helpers. Because the number of helpers varies, this fluctuation may look like social effects from the helpers. Likewise, individuals may differ in their effort depending on their breeding role (parent or helper). To investigate these possibilities, we created random regression models [9] of bird ID × role and bird ID × helper count, compared their fit to a model with a social current environment effect (model 4.2), and examined whether the presence of individual plasticity reduced or eliminated the social current environment variance.

Starting with the social effects model

$$z_{iyk} = \text{fixed effects} + E_{D,i} + C_{D,iy} + A_{D,i} + \sum E_{S,j} + \sum C_{S,jy} + \sum A_{S,j} + N_{my} + E_{iyk} \quad \text{(Model 2D)}$$

we fit a random regression with breeding role

$$z_{iyk} = \text{fixed effects} + E_{D,i} + \text{role} \cdot F_{D,i} + C_{D,iy} + A_{D,i} + \sum E_{S,j} + \sum C_{S,jy} + \sum A_{S,j} + N_{my} + E_{iyk}$$
(Model 2E)

where role is coded -1 for parents and 1 for helpers and $F_{D,i}$ is a random slope effect for each individual. Model 5.2 did not improve in terms of fit over model 4.2 (LR = 1.44, df = 1, p = .23). The REML estimate of the social current environment variance was also the same in both models (component = .0779, se = .0170). The slope variance was not significant (component = .026, se = .024).

We then fit individual slopes for number of helpers

$$z_{iyk} = \text{fixed effects} + E_{D,i} + \text{helpers} \cdot G_{D,i} + C_{D,iy} + A_{D,i} + \sum E_{S,j} + \sum C_{S,jy} + \sum A_{S,j} + N_{my} + E_{iyk}$$
(Model 2F)

where 'helpers' is the number of helpers and $G_{D,i}$ is the individual slope coefficients. The group size plasticity model was also not significantly better (LR = 3.85, df = 1, p = .05) and the social current environment variance component was only slightly reduced (.0725, se = .0170) and the slope variance was not significant (component = .016, se = .011).

8 SEX DIFFERENCES IN REPEATABILITY

A study of house sparrows *Passer domesticus* [10] found that between- and within-year repeatabilities were higher in males than females, so we tested for sex differences in variance using data on parents.

Starting with a model that fit direct genetic, permanent environment, current environment, and nest effects

$$z_{iyk} = \text{fixed effects} + E_{D,i} + C_{D,iy} + A_{D,i} + N_{my} + E_{iyk}$$ (Model 1A)

We first estimated separate residual variances for each sex and found some improvement in model fit (LR = 3.88, df = 1, p = .049). The residual variance for females (.037, se = .02) was higher than that for males (.32, se = .02) but were not substantially different from each other ($z = (.37 - .32) / \surd\ (.02^2 + .02^2) = 1.96$, p = .05).

A model that retained sex-specific residual variances and also fit separate permanent environment had convergence issues where the REML estimates of the permanent environment effects both went to zero.

Fitting sex-specific current environment variances did not improve model fit (LR = .49, df = 1, p = .48). We also did not find any evidence for sex differences in genetic variance in a model that also fit a genetic covariance term (LR = .41, df = 2, p = .81). The genetic covariance between male and female provisioning rates was r_G = .98.

9 SEX DIFFERENCES IN SOCIAL EFFECTS

Because male long-tailed tits reduce effort more in response to helpers then females do [11], it is possible that there is a sex difference in sensitivities to social effects. To test this we fit a series of categorical random interaction models where an individual's social effect varied either as a function of its own sex or its partner's sex.

Starting from the basic social effects model, a focal individual i's phenotype y_i is a result of its direct effect on itself, D_i, and its J partners' social effects, S_j

$$y_i = \mu + D_i + \sum_j S_j + e_i \qquad \text{(S1)}$$

In modelling social effects separately by sex, we can consider social effects being moderated either by the sex of the focal individual (i) or by the sex of its partner (individual j).

If social effects differ depending on the sex of the target then we are positing that individuals of one sex more extremely increase and decrease their feeding rate in response to their partners' presence.

$$y_i = \mu + D_i + \sum_j S_{j,\text{sex}_i} + e_i \qquad \text{(S2)}$$

where $S_{j,\text{sex}_i}$is individual j's effect on the phenotype of individual i depending on the sex of individual i. That is, individual j has one social effect on females and another social effect on males. Since in a cooperative breeding context where birds are interacting in groups rather than in pairs, this sex effect would have to be a difference in females' and males' behaviors in response to individual j rather than a difference in individual j's behavior when

interacting with a female or male partner. In this model separate variance components are fit for a partner's effects on female focals $S_{j,\text{female}_i} \sim \text{Norm}(0, V_{S,\text{female}_i})$ and on male focals $S_{j,\text{female}_i} \sim \text{Norm}(0, V_{S,\text{male}_i})$. Given previous findings that males reduce effort in the presence of helpers more than females do [11], if there is a difference then we would expect that males would be the more responsive sex.

If instead social effects differ depending on the sex of the partner, then we are positing that males and females differ in how much they are responded to. Because we are fitting effects to individuals, this is different from saying the mean response differs between male and female partners. Instead, if one sex varies more in the quality and quantity of provisioning behavior, then partners will respond more extremely to individuals of that sex. The model is

$$y_i = \mu + D_i + \sum_j S_{j,\text{sex}_j} + e_i \qquad \text{(S3)}$$

where the only difference from equation S2 is that the social effect is now being indexed by the sex of individual j (the partner) rather than the sex of the focal individual i. Because a bird only has one sex, it is not possible to estimate the individual-level covariance between these components.

We built models to test sex differences in social effects. Given that in our main result we detected environmental but not genetic social effects, we fit models with sex-specific social environment effect variances. We started from the sex-specific repeatability model from section 7 (above). This model had single direct genetic, direct permanent environment, direct current environment, and nest effects. The model also had sex-specific residuals.

We tested for sex-specific social permanent environment effects and sex-specific social current environment effects using either the sex of the focal individual or its partners as the moderating variables. In each case we use the social permanent or social current effects as a base line, *V(social)*, and compare models that fit separate *V(social female)* or *V(social male)* using the sex of the focal or the sex of the partners. We test for the significance of the sex × social variances against the baseline using the likelihood ratio test and compare all three sets of models using weighted AIC [12], which is the probability of the

model given the data (among the models being compared). Variance components are listed with standard errors.

Table S2. Sex random interaction models for social effects comparing interaction with the sex of the focal individual or the sex of the partner versus a baseline model with a single social effect V(Social), for social permanent environment or social current environment effects. V(social female) = variance of social female effects, V(social male) = variance of social male effects, COV(sex) = environmental covariance between female and male effects, LR = likelihood ratio of model versus baseline, df = degrees of freedom, p = p value from a likelihood ratio test, AIC_w = weighted Akaike Information Criterion.

Social permanent E	V(social)	V(social female)	V(social male)	COV(sex)	LR	df	p	AIC_w
Baseline	0.042 ± 0.012	---	---	---	1	---	---	.25
Sex of focal	---	0.060 ± 0.022	0.047 ± 0.018	0.026 ± 0.015	5.7	2	0.055	.61
Sex of partner	---	0.055 ± 0.021	0.034 ± 0.013	---	0.8	1	0.37	.14

Social current E	V(social)	V(social female)	V(social male)	COV(sex)	LR	df	p	AIC_w
Baseline	0.078 ± 0.017	---	---		1	---	---	.32
Sex of focal	---	0.088 ± 0.025	0.087 ± 0.020	0.060 ± 0.020	5.1	2	0.080	.54
Sex of partner	---	0.089 ± 0.026	0.071 ± 0.019	---	0.4	1	0.54	.14

We found that permanent social effects did not differ based on the sex of the target ($p = 0.055$) or the partners ($p = 0.37$). The correlation between permanent social focal-sex effects was 0.49. Current social effects also did not differ based on the sex of the target ($p = 0.080$) or the partners ($p = 0.54$). The correlation between current social focal-sex effects was 0.68. Thus we found no evidence that social effects differ between males and females.

10 EFFECT OF RELATEDNESS ON SOCIAL EFFECTS

When kin and nonkin interact, it is also possible that the social effects between individuals differ depending on their relatedness [13]. We explored this possibility by fitting social effects moderated by the relatedness between target and partner individuals

$$y_i = \mu + D_i + \sum_j S_{j,\mathrm{kin}_{ij}} + e_i \qquad \text{(S4)}$$

where kin_{ij} codes the relatedness between individuals *i* and *j* as either 'kin' or 'strangers'. We fit separate variance components for the two relatedness categories as well as the covariance between them. We fit categorical random interaction models using both permanent and current social environment effects. Estimating separate variances components for the two relatedness categories did not improve model fit for either the permanent (LR = 2.8, df=2, $p = .24$) or current (LR = 2.6, df = 2, $p = .28$) social environment models, and thus the data did not support a difference in the size of social effects between kin and nonkin. The correlation between direct and indirect effects was $r = .93$ in the permanent social environment model and $r = .78$ in the temporary social environment model.

11 PHENOTYPIC PLASTICITY AS POSSIBLE CONFOUND

In our main analysis we separated out current and permanent environmental sources of social effects because group composition varies with a breeding season, with helpers joining breeding pairs and failed breeders becoming helpers at varying points in the breeding process. These changes over time

create the possibility that individual differences in plasticity [14] to time-varying factors could create the appearance of social effects. In other words, a bird could vary its behaviour in response to a changing factor but this change coincides with the arrival of a helper and thus the change could appear to be a social effect of the helper.

Two such time varying factors that were measured and that change feeding rates are brood age and number of helpers. In the main analysis we fit these variables with constant slopes (that is, as fixed effects). We fitted varying slopes by individual ID (continuous random interaction) and then test whether the inclusion of social effects still significantly improve model fit.

Starting from a model that contained only direct effects, we added varying slopes for brood age and number of helpers. Parents did not vary in their plasticity to number of helpers (LR = 0.20, df = 1, $p = 0.66$) but they did vary in plasticity to brood age (LR = 18, df = 1, $p < 0.001$). Parental plasticity to brood age accounted for about 1% of the phenotypic variance. By comparison, average plasticity to brood age explained 23% of the phenotypic variance in feeding rates. We then added a social environment effect to the random slopes model. The social environment variance still significantly improved model fit (LR = 6.4, df = 1, $p = 0.01$).

12 GENOTYPING

Genomic DNA was extracted from blood (stored in absolute ethanol) using ammonium acetate [15, 16]. Nineteen published microsatellite loci were combined with two sex-typing markers and arranged into three multiplex (MP) sets using MULTIPLEX MANAGER v1.2 [17], each set containing between six and eight markers (Table S1). The inclusion of two sex-typing markers enabled the confident assignment of sex and allowed us to identify sample mix-ups [P2D-P8 and Z002A: 18, 19, 20]. PCR was performed in a 2-μl multiplex reaction containing 10 ng of dried genomic DNA, 1 μl of QIAGEN Multiplex PCR Master Mix (containing HotStarTaq DNA polymerase), and the forward and reverse primers [following 21 the final concentrations of the primers are provided in Table S1]. Amplification was performed using a DNA Engine Tetrad PTC-225 thermal cycler (MJ Research, Bio-Rad, Hemel

Hempstead, Herts., UK). The PCR cycling conditions were as follows: an initial denaturation at 95 °C for 15 min, followed by 33 cycles of 94 °C for 30 s, 56°C (MP set 1 and set 2) or 58°C (MP set3) for 90 s, 72°C for 60 s, and a final 30 min at 60°C. Amplified products were loaded on to the ABI3730 48-well capillary DNA Analyser (Applied Biosystems, California, USA) and allele sizes assigned using GENEMAPPER v3.7 (Applied Biosystems, California, USA).

The genotypes of 32 unrelated individuals belonging to a single population located in the Rivelin Valley were used to characterise the loci. Expected and observed heterozygosities and the estimated null allele frequency of each locus was calculated using CERVUS v3.0.3 [22]. For all 19 microsatellite loci, both sexes amplified and a proportion of the females were heterozygous indicating that all loci were autosomal in this species. Tests for departure from Hardy –Weinberg equilibrium and assessment of linkage disequilibrium were performed using GENEPOP v4.2 [23, 24] and a False Discovery Rate (FDR) correction for multiple tests applied [25]. After FDR correction, no locus deviated from Hardy–Weinberg equilibrium and no groups of loci displayed linkage disequilibrium.

13 PEDIGREE CONSTRUCTION

We used FRANz [26] to reconstruct the whole pedigree using the molecular markers. We first ran FRANz using prior information about individual birth-death events and genetically-determined sex to identify all likely parents (LOD > 0) of each individual. For each individual we excluded their social mother if she had been genotyped and was not matched as one of the parents. We output a pedigree of genetically-matched mother-offspring pairs and reran FRANz using this information to match fathers and to find full sibling groups among the founders and immigrants. The extra-pair paternity rate was 3% of nestlings. FRANz identified 20 likely full sibling groups out of the birds with unknown parentage. For these full sibling groups we entered dummy parent IDs into the pedigree. The full sibling test also identified 21 likely full sibling groups for whom only one parent was known from the social pedigree

but the missing parent was not found among the genotyped candidates. For these groups we created a dummy ID for the missing parent in the pedigree. For ungenotyped individuals we used parents assigned from the social pedigree.

REFERENCES

[1] Nam, K.-B., Simeoni, M., Sharp, S.P. & Hatchwell, B.J. 2010 Kinship affects investment by helpers in a cooperatively breeding bird. *Proc Biol Sci* **277**, 3299-3306.
[2] Wilson, A.J. 2008 Why h[2] does not always equal V[A]/V[P]? *J Evol Biol* **21**, 647-650. (doi:10.1111/j.1420-9101.2008.01500.x).
[3] MacColl, A.D. & Hatchwell, B.J. 2003 Heritability of parental effort in a passerine bird. *Evolution* **57**, 2191-2195.
[4] Gelman, A. 2005 Analysis of variance—why it is more important than ever. *Ann Stat* **33**, 1-53.
[5] Bijma, P. 2013 The quantitative genetics of indirect genetic effects: a selective review of modelling issues. *Heredity*, 1-9. (doi:10.1038/hdy.2013.15).
[6] Bijma, P. 2011 A general definition of the heritable variation that determines the potential of a population to respond to selection. *Genetics* **189**, 1347-1359.
[7] Bijma, P. 2010 Multilevel selection 4: modeling the relationship of indirect genetic effects and group size. *Genetics* **186**, 1029-1031.
[8] Morrissey, M.B. & Wilson, A.J. 2010 pedantics: an R package for pedigree-based genetic simulation and pedigree manipulation, characterization and viewing. *Mol Ecol Resour* **10**, 711-719. (doi:10.1111/j.1755-0998.2009.02817.x).
[9] Martin, J.G.A., Nussey, D.H., Wilson, A.J. & Réale, D. 2011 Measuring individual differences in reaction norms in field and experimental studies: a power analysis of random regression models. *Methods Ecol Evol* **2**, 362-374.
[10] Nakagawa, S., Gillespie, D., Hatchwell, B. & Burke, T. 2007 Predictable males and unpredictable females: sex difference in repeatability of parental care in a wild bird population. *J Evol Biol* **20**, 1674-1681.
[11] Meade, J., Nam, K.-B., Beckerman, A.P. & Hatchwell, B.J. 2010 Consequences of 'load-lightening' for future indirect fitness gains by helpers in a cooperatively breeding bird. *J Anim Ecol* **79**, 529-537.

[12] Burnham, K.P. & Anderson, D.R. 2002 *Model selection and multimodel inference : a practical information-theoretic approach*. 2nd ed. New York, Springer; xxvi, 488 p. p.
[13] Alemu, S.W., Berg, P., Janss, L. & Bijma, P. 2013 Indirect genetic effects and kin recognition: estimating IGEs when interactions differ between kin and strangers. *Heredity* **112**, 197-206.
[14] Nussey, D., Wilson, A. & Brommer, J. 2007 The evolutionary ecology of individual phenotypic plasticity in wild populations. *J Evol Biol* **20**, 831-844.
[15] Nicholls, J.A., Double, M.C., Rowell, D.M. & Magrath, R.D. 2000 The evolution of cooperative and pair breeding in thornbills Acanthiza (Pardalotidae). *J Avian Biol* **31**, 165-176. (doi:10.1034/j.1600-048X.2000.310208.x).
[16] Richardson, D.S., Jury, F.L., Dawson, D.A., Salgueiro, P., Komdeur, J. & Burke, T. 2000 Fifty Seychelles warbler (Acrocephalus sechellensis) microsatellite loci polymorphic in Sylviidae species and their cross-species amplification in other passerine birds. *Mol Ecol* **9**, 2225-2230. (doi:10.1046/j.1365-294X.2000.105338.x).
[17] Holleley, C.E. & Geerts, P.G. 2009 Multiplex Manager 1.0: a cross-platform computer program that plans and optimizes multiplex PCR. *Biotechniques* **46**, 511.
[18] Dawson, D.A., Horsburgh, G.J., Krupa, A.P., Stewart, I.R., Skjelseth, S., Jensen, H., Ball, A.D., Spurgin, L.G., MANNARELLI, M.E. & Nakagawa, S. 2012 Microsatellite resources for Passeridae species: a predicted microsatellite map of the house sparrow Passer domesticus. *Mol Ecol Resour* **12**, 501-523.
[19] Griffiths, R., Double, M.C., Orr, K. & Dawson, R.J.G. 1998 A DNA test to sex most birds. *Mol Ecol* **7**, 1071-1075.
[20] Dawson, D.A. 2007 Genomic analysis of passerine birds using conserved microsatellite loci. Sheffield, UK, University of Sheffield.
[21] Kenta, T., Gratten, J., Haigh, N., Hinten, G., Slate, J., Butlin, R. & Burke, T. 2008 Multiplex SNP-SCALE: a cost-effective medium-throughput single nucleotide polymorphism genotyping method. *Mol Ecol Resour* **8**, 1230-1238.
[22] Kalinowski, S.T., Taper, M.L. & Marshall, T.C. 2007 Revising how the computer program CERVUS accommodates genotyping error increases success in paternity assignment. *Mol Ecol* **16**, 1099-1106.
[23] Raymond, M. & Rousset, F. 1995 GENEPOP (version 1.2): population genetics software for exact tests and ecumenicism. *J Heredity* **86**, 248-249.
[24] Rousset, F. 2008 genepop'007: a complete re-implementation of the genepop software for Windows and Linux. *Mol Ecol Resour* **8**, 103-106.
[25] Benjamini, Y. & Hochberg, Y. 1995 Controlling the false discovery rate: a practical and powerful approach to multiple testing. *J R Stat Soc Series B Stat Methodol*, 289-300.
[26] Riester, M., Stadler, P.F. & Klemm, K. 2009 FRANz: reconstruction of wild multi-generation pedigrees. *Bioinformatics* **25**, 2134-2139.
[27] Dawson, D.A., Ball, A.D., Spurgin, L.G., Martín-Gálvez, D., Stewart, I.R., Horsburgh, G.J., Potter, J., Molina-Morales, M., Bicknell, A.W. & Preston, S.A. 2013 High-utility conserved avian microsatellite markers enable parentage and population studies across a wide range of species. *BMC Genomics* **14**, 176.

[28] Dawson, D.A., Horsburgh, G.J., Küpper , C., Stewart, I.R.K., Ball, A.D., Durrant, K.L., Hansson, B., Bacon, I.D.A., Bird, S. & Klein, A. 2010 New methods to identify conserved microsatellite loci and develop primer sets of high cross-species utility–as demonstrated for birds. *Mol Ecol Resour* **10**, 475-494.
[29] Primmer, C., Møller, A. & Ellegren, H. 1996 A wide-range survey of cross-species microsatellite amplification in birds. *Mol Ecol* **5**, 365-378.
[30] Saladin, V., Bonfils, D., Binz, T. & Richner, H. 2003 Isolation and characterization of 16 microsatellite loci in the Great Tit Parus major. *Mol Ecol Notes* **3**, 520-522.
[31] Martinez, J., Soler, J., Soler, M., Moller, A. & Burke, T. 1999 Comparative population structure and gene flow of a brood parasite, the great spotted cuckoo (Clamator glandarius), and its primary host, the magpie (Pica pica). *Evolution*, 269-278.
[32] Piertney, S.B., MacColl, A.D.C., Bacon, P.J. & Dallas, J.F. 1998 Local genetic structure in red grouse (Lagopus lagopus scoticus): evidence from microsatellite DNA markers. *Mol Ecol* **7**, 1645-1654.
